# Healthy eating markers among adolescents from the municipal school system in Pelotas, Rio Grande do Sul, Brazil, 2019: a cross-sectional study

**DOI:** 10.1590/S2237-96222023000200019

**Published:** 2023-08-25

**Authors:** Jéssica Gularte Domingues, Francine Silva dos Santos, Cristina Corrêa Kaufmann, Ludmila Correa Muniz, Renata Moraes Bielemann, Gicele Costa Mintem

**Affiliations:** 1 Universidade Federal de Pelotas, Programa de Pós-Graduação em Nutrição e Alimentos, Pelotas, RS, Brazil; 2 Universidade de São Paulo, Núcleo de Pesquisas Epidemiológicas em Nutrição e Saúde, São Paulo, SP, Brazil

**Keywords:** Students, Diet, Healthy, Eating, Feeding Behavior, Cross-Sectional Studies, Estudiantes, Dieta Saludable, Consumo Alimentario, Comportamiento Alimentario, Estudios Transversales, Estudantes, Dieta Saudável, Consumo Alimentar, Comportamento Alimentar, Estudos Transversais

## Abstract

**Main results:**

Around 3% of the students regularly consumed the five healthy eating markers, with beans being the most frequently consumed; strong association was found between healthy eating markers and behavioral variables.

**Implications for services:**

The results obtained can contribute for interventions, programs and actions, particularly in the school environment, aimed at promoting an adequate and healthy diet.

**Perspectives:**

Better targeting of interventions, programs and actions developed in the school environment, seeking to increase consumption of fresh and minimally processed food, along with the adoption of healthy behaviors.

## Abstract (key points)

**Objective:**

to analyze the consumption of healthy eating markers among adolescent students in Pelotas, Rio Grande do Sul, Brazil, in 2019.

**Methods:**

this was a cross-sectional study carried out in schools taking part in the Health at School Program. Consumption of the five markers (beans, milk, fruit, cooked vegetables and raw salads) within five days prior to the study, obtained through a self-administered questionnaire, was defined as regular. Association between demographic, socioeconomic and behavioral variables and the outcomes was analyzed using Poisson regression.

**Results:**

among the 797 respondents, 2.8% (95%CI 1.8;4.1) reported regular consumption. Beans were the most regularly consumed food (48.3%); fruit, cooked vegetables and raw salads were consumed regularly by less than 30% of the students. Doing physical activities, having breakfast and having meals with the family were associated with regular consumption of the foods assessed.

**Conclusion:**

regular consumption of healthy foods was low in this population, and was associated with behavioral factors.

## Resumo

**Objetivo:**

analisar o consumo de marcadores de alimentação saudável entre adolescentes escolares de Pelotas, Rio Grande do Sul, Brasil, em 2019.

**Métodos:**

estudo transversal, realizado nas escolas vinculadas ao Programa Saúde na Escola; o consumo dos cinco marcadores (feijão, leite, frutas, vegetais cozidos, saladas cruas) em até cinco dias antecedentes ao estudo, obtido mediante questionário autoaplicado, foi definido como regular; a associação de variáveis demográficas, socioeconômicas e comportamentais com os desfechos foi analisada por regressão de Poisson.

**Resultados:**

entre os 797 entrevistados, 2,8% (IC_95%_ 1,8;4,1) referiram consumo regular; o feijão foi o alimento mais consumido regularmente (48,3%); frutas, vegetais cozidos e saladas cruas foram consumidos regularmente por menos de 30% dos escolares; praticar atividade física, tomar café da manhã e realizar refeições com a família estiveram associados ao consumo regular dos alimentos avaliados.

**Conclusão:**

o consumo regular de alimentos saudáveis, encontrado baixo nessa população, associou-se com fatores comportamentais.

## Resumen

**Objetivo:**

analizar el consumo de marcadores de alimentación saludable entre estudiantes adolescentes de Pelotas, Rio Grande do Sul, Brasil, en 2019.

**Métodos:**

estudio transversal realizado en escuelas vinculadas al Programa Salud en la Escuela. Se definió como regular el consumo de los cinco marcadores (porotos, leche, frutas, vegetales cocidos y ensaladas crudas) dentro de los cinco días previos al estudio, obtenidos a través de un cuestionario autoadministrado. La asociación entre variables demográficas, socioeconómicas y conductuales con los resultados se analizó mediante la regresión de Poisson.

**Resultados:**

entre los 797 encuestados, el 2,8% (IC95% 1,8; 4,1) informó un consumo regular. Los porotos fueron el alimento más consumido (48,3%). Frutas, verduras cocidas y ensaladas crudas fueron consumidas regularmente por menos del 30% de los estudiantes. La práctica de actividad física, el desayuno y las comidas en familia se asociaron con mayor consumo de los alimentos evaluados.

**Conclusión:**

el consumo regular de alimentos saludables fue bajo en esta población y se asoció con factores conductuales.

## INTRODUCTION

According to the World Health Organization (WHO), adolescence is the stage of life between the ages of 10 and 19, corresponding to the transition from childhood to adulthood.^1^ This is an age group considered to be at nutritional risk, given the profound biological, social and emotional changes that adolescents go through. Accelerated physical growth and psychosocial development, particularly during puberty, as well as intense cognitive stimulation, increase demand for energy and nutrients.^2^ In this way, adequate nutrition at this age contributes to achieving the expected parameters of growth and development, as well as to the prevention of chronic diseases in adult life.^3^

An adequate and healthy diet should be based on the consumption of fresh and minimally processed foods, such as beans, fruit and vegetables, which are an important factor in the protection of health and well-being.^4^ High consumption of ultra-processed foods (rich in fat, sugar and sodium), insufficient consumption of fresh foods and a predominance of sedentary behaviors has been noted among adolescents.^5^ ^,^ ^6^ The influence of family, friends, school, marketing and advertising, as well as economic and cultural factors, can determine adolescents’ eating choices and behavior.^7^ It should be noted that during childhood and adolescence, habits and lifestyles are established that tend to remain in adult life, making it essential to carry out preventive actions and promote healthy habits in these stages of life.^8^

Schools are an environment with a strong influence on the education of students and a privileged space for the development of health promotion and disease prevention actions among children and adolescents. For this reason, several programs and public policies are carried out in schools. In terms of health and food and nutrition security for schoolchildren, the National School Meals Program (*Programa Nacional de Alimentação Escolar* - PNAE) stands out, with the purpose of contributing to growth and biopsychosocial development, learning, school performance and promoting healthy eating habits among students, through food and nutrition education actions, in addition to offering meals at school.^9^

The Brazilian public education network is benefited by the Health at School Program (*Programa Saúde na Escola* - PSE), an intersectoral policy coordinated by the ministries of health and education, developed with the objective of contributing to the integral formation of students through disease prevention and health care promotion actions, with a view to addressing vulnerabilities that compromise the full development of children, adolescents and young people.^10^ Activities to promote adequate and healthy nutrition and prevent obesity are among the actions developed by the PSE.^10^

Considering the importance of healthy eating for proper growth and development during adolescence, as well as for the prevention of chronic noncommunicable diseases (NCDs), assessment of food consumption in the school environment is essential for guiding food and nutrition actions and policies.

The aim of this study was to analyze the consumption of healthy eating markers among adolescents in the 9^th^ grade of elementary school in the municipal education network of Pelotas, in the state of Rio Grande do Sul, Brazil.

## METHODS

This was a cross-sectional, school-based study carried out in schools taking part in the PSE in the municipal education network of Pelotas. During the study period, the urban area of ​​the city had 40 municipal schools, 30 of which provided complete elementary education (25 of these were taking part in the PSE), where we chose to conduct our research, and a further 10 that provided incomplete elementary education (9 of these were taking part in the PSE). Students enrolled at schools where the PSE is in place participate in the Disease Prevention and Health Promotion Program (*Programa de Prevenção de Doenças e Promoção da Saúde*), which is the main reason why our research was done at these schools. Data collection was carried out between April and December 2019, but had to be interrupted at the beginning of the 2020 school year, given the need to suspend academic and school activities due to the COVID-19 pandemic. As such, data collection was restricted to the 25 elementary primary and middle schools taking part in the PSE. Of the 11,658 students enrolled at these schools, all students enrolled in the 9^th^ grade were considered eligible.

The data collection instrument consisted of a modified version of the National School Health Survey (*Pesquisa Nacional de Saúde do Escolar* - PeNSE) questionnaire.^11^ The questionnaire was self-administered, that is, filled in by the students themselves in the classroom, during the research team visits to the schools. Students with any physical or mental disability that prevented them from answering the questionnaire were excluded. Students not found in the classroom after three attempts by the research team, on different days and times, were considered to be losses.

The main PeNSE questionnaire had 12 blocks of questions. In order to assess the outcomes, questions from the block related to food were used, aiming to identify the frequency of consumption of certain foods or beverages in the seven days prior to the day the questionnaire was administered. This block of questions consisted of ten food consumption markers (five healthy eating markers and five unhealthy eating markers). For this study, the five healthy eating markers were used: beans; greens and cooked vegetables (vegetables in general); fresh fruit (or fruit salad); raw salads; milk (or milk-based products such as yogurt).

The outcomes analyzed were: (i) the proportion of adolescents who regularly consumed each food item (period ≥ 5 days, in the seven days prior to the study);^12^ and (ii) the food item consumption score, calculated by assigning points to each healthy eating marker, ranging from 0 (consumption between 0 and 2 days/week), 1 (consumption 3 to 4 days/week) and 2 (consumption 5 days or more a week), with a minimum score of 0 and a maximum score of 10 points.^13^

For description purposes, the percentage distribution of the weekly frequency of consumption of each food item was analyzed (did not eat the item; ate it 1 day, 2, 3, 4, 5, 6 or 7 days).

The following independent variables were analyzed:

a) Demographic and socioeconomic variables

- sex (male; female);
- race/skin color (White; brown-Black; yellow-Indigenous);
- age group (at last birthday: up to 14; 15; 16 or more); and
- maternal schooling (completed years of study: up to 8; 9-11; 12 or more).

b) Behavioral variables

- physical exercise (physical active; physically inactive), where the physically active were considered to be those who did at least 300 minutes of physical activities in their leisure time, in the week prior to the interview;^14^
- habit of having breakfast (no; yes);
- eating meals in front of screens (no; yes); and
- having meals with the family (no; yes).

The data were double entered using the EpiData program, and analyzed using the Stata version 12.1 program. Absolute and relative frequency analyses were performed to describe the population we studied, stratified by sex. To measure the frequency of regular consumption of healthy eating markers, 95% confidence intervals (95%CI) were also estimated. Crude and multiple Poisson regression with adjustment for robust variance was used for the purpose of assessing associations between the outcome and the independent variables. The results were presented as prevalence ratios (PR), with their respective 95%CI.^15^

Independent variables were grouped into two levels: demographic and socioeconomic variables (level 1); and behavioral variables (level 2). In the adjusted analyses, the hierarchical conceptual model strategy was followed,^16^ for both levels mentioned. Variables were included in the model using backward selection, level by level, excluding variables with p-value > 0.20. Variables that had a p-value ≤ 0.20 in their level in the adjusted model were kept in the model.

The study project was approved by the Research Ethics Committee of the *Universidade Federal de Pelotas* Faculty of Nursing, as per Opinion No. 2.843.572/2018, issued on August 24, 2018. The carrying out of the survey was authorized by the Pelotas City Education and Sports Department. All participants were informed of the nature of the study and consulted about their willingness to participate, confirmed by signing an Informed Assent form, although only those whose parents or guardians signed an Informed Consent form answered the questionnaire.

## RESULTS

Of the total of 951 students in the 9^th^ grade of schools belonging to the Pelotas municipal education network taking part in the PSE, 810 agreed to participate in the study (11.3% losses and 3.6% refusals) and of these, 797 completely answered the food block part of the questionnaire and were included in the analyses (83.8% of eligible students).

Table 1 shows the characteristics of the participants. About 60.0% of the adolescents were children of mothers with nine or more years of schooling. The habit of having breakfast was reported by 57.0% of the participants. With regard to behavior during meals, 85.5% of adolescents had meals with their family while 79.9% ate their meals in front of screens. Fewer than one in five students reported practicing physical activity for a period equal to or greater than 300 minutes/week.

**Table 1:** - Demographic, socioeconomic and eating behavioral characteristics among 9^th^ grade municipal school students, Pelotas, Rio Grande do Sul, 2019

Variables	Total (n = 797)		Male (n = 386)		Female (n = 411)	
	n	%	n	%	N	%
Race/skin color^a^						
Yellow-Indigenous	24	3.1	13	3.4	11	2.7
White	479	60.9	241	63.6	238	58.5
Brown/Black	283	36.0	125	33.0	158	38.8
Age group (at last birthday)						
≤ 14	308	38.6	138	35.8	170	41.4
15	333	41.8	165	42.7	168	40.8
≥ 16	156	19.6	83	21.5	73	17.8
Maternal schooling (completed years of study)^b^						
≤ 8	255	41.0	102	35.3	153	46.1
9-11	227	36.6	114	39.4	113	34.0
≥ 12	139	22.4	73	25.3	66	19.9
Physical exercise (minutes/week)^c^						
< 300	639	82.8	271	73.0	368	92.0
≥ 300	132	17.2	100	27.0	32	8.0
Habit of having breakfast^d^						
No	330	43.0	136	36.7	194	49.0
Yes	437	57.0	235	63.3	202	51.0
Eating meals in front of screens^e^						
No	136	20.1	60	18.0	76	22.1
Yes	542	79.9	274	82.0	268	77.9
Having meals with the family^f^						
No	115	14.5	62	16.2	53	13.0
Yes	677	85.5	322	83.8	355	87.0

a) n = 786; b) n = 621; c) n = 771; d) n = 767; e) n = 678; f) n = 792.

Of the total 797 participants, only 2.8% (95%CI 1.8;4.1) regularly consumed the five healthy eating markers assessed, of whom 3.1% (95%CI 1.8;5.4) were male and 2.4% (95%CI 1.3;4.5) were female.

**Figure 1:**
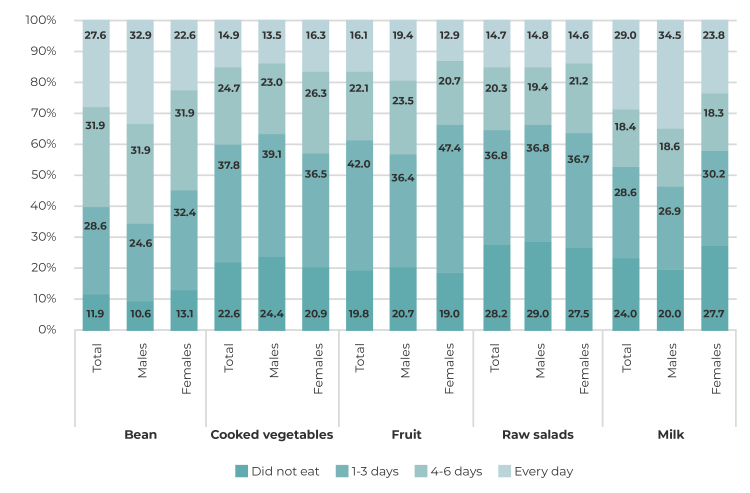
- Distribution of frequency of consumption of healthy eating markers in the week prior to the survey, among 9^th^ grade municipal school students (n = 797), Pelotas, Rio Grande do Sul, 2019

Figure 1 shows the frequency of consumption of healthy eating markers by the total study population, according to sex. The food item with the highest frequency of regular consumption by adolescents was beans (48.3%), followed by milk (40.4%). Among males, beans were consumed regularly by 54.7% (95%CI 49.6;59.6), and milk by 46.7% (95%CI 41.7;51.7). Regular consumption of fruit, cooked vegetables and raw salads was reported, respectively, by 29.1% (95%CI 26.1;32.4), 28.6% (95%CI 25.6;31.9) and 27.3% (95%CI 24.2;30.4) of the participants (Figure 1).

Table 2 presents the results of the crude analysis of association of demographic, socioeconomic and behavioral factors with the regular consumption of healthy eating markers. After adjustment, according to the conceptual model (Table 3), being of the female sex was associated with lower regular consumption of beans (PR = 0.78; 95%CI 0.68;0.90), milk (PR = 0.74; 95%CI 0.60;0.90) and fruit (PR = 0.74; 95%CI 0.60;0.93); however, female adolescents reported regular consumption of cooked vegetables 39.0% higher than the consumption of this same marker among males.

**Table 2:** - Crude analysis of factors associated with consumption of healthy eating markers among 9^th^ grade municipal school students (n = 797), Pelotas, Rio Grande do Sul, 2019

Variables	Beans	Milk	Fruit	Raw salads	Cooked vegetables
	PR^a^ (95%CI^b^)	PR^a^ (95%CI^b^)	PR^a^ (95%CI^b^)	PR^a^ (95%CI^b^)	PR^a^ (95%CI^b^)
1^st^ level: demographic variables and socioeconomic variable					
Sex					
Male	1.00	1.00	1.00	1.00	1.00
Female	0.77 (0.67;0.90)^c^	0.74 (0.62;0.88)^c^	0.74 (0.59;0.92)^c^	1.12 (0.89;1.41)	1.36 (1.09-1.71)^c^
Race/skin color					
Yellow-Indigenous	1.43 (1.01;1.98)^c^	1.02 (0.65;1.59)	1.77 (1.16;2.71)^c^	1.57 (0.96;2.58)	1.65 (1.04;2.61)^c^
White	1.00	1.00	1.00	1.00	1.00
Brown/Black	1.24 (1.07;1.44)^c^	0.72 (0.59;0.88)^c^	1.02 (0.80;1.28)	1.05 (0.83;1.34)	1.02 (0.80;1.29)
Age group (at last birthday)					
≤ 14	1.00	1.00	1.00	1.00	1.00
15	1.07 (0.91;1.26)	0.83 (0.68;1.03)	1.23 (0.97;1.56)	1.12 (0.87;1.43)	1.08 (0.85;1.38)
≥ 16	1.21 (1.00;1.46)^c^	0.79 (0.58;1.06)	0.99 (0.72;1.36)	0.87 (0.62;1.22)	0.89 (0.64;1.25)
Maternal schooling (completed years of study)					
≤ 8	1.00	1.00	1.00	1.00	1.00
9-11	0.86 (0.71;1.04)	1.20 (0.95;1.52)	1.09 (0.83;1.44)	1.07 (0.80;1.43)	1.03 (0.79;1.36)
≥ 12	0.92 (0.74;1.15)	1.49 (1.17;1.90)^c^	1.07 (0.78;1.47)	1.03 (0.73;1.44)	1.06 (0.78;1.45)
**2^nd^ level: behavioral variables**					
Physical exercise (minutes/week)					
< 300	1.00	1.00	1.00	1.00	1.00
≥ 300	1.23 (1.03;1.45)^c^	1.35 (1.11;1.64)^c^	20.8 (1.67;2.58)^c^	1.42 (1.09;1.86)^c^	1.54 (1.20;1.96)^c^
Habit of having breakfast					
No	1.00	1.00	1.00	1.00	1.00
Yes	1.38 (1.18;1.62)^c^	1.52 (1.26;1.83)^c^	1.36 (1.08;1.72)^c^	1.26 (0.99;1.59)	1.34 (1.06;1.70)^c^
Eating meals in front of screens					
No	1.00	1.00	1.00	1.00	1.00
Yes	0.85 (0.71;1.02)	0.99 (0.79;1.23)	0.84 (0.64;1.10)	0.85 (0.64;1.13)	0.67 (0.52;0.86)^c^
Having meals with the family					
No	1.00	1.00	1.00	1.00	1.00
Yes	1.35 (1.05;1.73)	0.96 (0.76;1.22)	1.02 (0.75;1.40)	1.15 (0.81;1.62)	1.21 (0.86;1.71)

n = 786; b) n = 621; c) n = 771; d) n = 767; e) n = 678; f) n = 792.

**Table 3:** - Adjusted analysis of factors associated with consumption of healthy eating markers among 9^th^ grade municipal school students (n = 797), Pelotas, Rio Grande do Sul, 2019

Variables	Beans	Milk	Fruit	Raw salads	Cooked vegetables
	PR^a^ (95%CI^b^)	PR^a^ (95%CI^b^)	PR^a^ (95%CI^b^)	PR^a^ (95%CI^b^)	PR^a^ (95%CI^b^)
1^st^ level: demographic variables and socioeconomic variable					
Sex					
Male	1.00	1.00	1.00	1.00	1.00
Female	0.78 (0.68;0.90)^c^	0.74 (0.60;0.90)^c^	0.74 (0.60;0.93)^c^	1.12 (0.89;1.41)	1.39 (1.11;1.75)^c^
Race/skin color					
Yellow-Indigenous	1.41 (1.01;1.98)^c^	1.11 (0.61;2.04)	1.78 (1.18;2.67)^c^	1.57 (0.96;2.58)	1.67 (1.05;2.66)^c^
White	1.00	1.00	1.00	1.00	1.00
Brown/Black	1.26 (1.09;1.46)^c^	0.80 (0.65;1.00)	1.04 (0.82;1.31)	1.05 (0.83;1.34)	1.00 (0.79;1.26)
Age group (at last birthday)					
≤ 14	1.00	1.00	1.00	1.00	1.00
15	1.03 (0.85;1.25)	0.83 (0.68;1.03)	1.17 (0.92;1.50)	1.10 (0.86;1.42)	1.08 (0.85;1.38)
≥ 16	1.08 (0.86;1.36)	0.79 (0.58;1.06)	0.91 (0.66;1.27)	0.84 (0.59;1.18)	0.89 (0.64;1.25)
Maternal schooling (completed years of study)					
≤ 8	1.00	1.00	1.00	1.00	1.00
9-11	0.86 (0.71;1.04)	1.12 (0.89;1.42)	1.08 (0.82;1.43)	1.13 (0.84;1.52)	1.09 (0.83;1.43)
≥ 12	0.91 (0.73;1.14)	1.36 (1.06;1.74)^c^	1.08 (0.77;1.50)	1.09 (0.77;1.53)	1.11 (0.81;1.52)
**2^nd^ level: behavioral variables**					
Physical exercise (minutes/week)					
< 300	1.00	1.00	1.00	1.00	1.00
≥ 300	1.13 (0.94;1.35)	1.41 (1.13;1.77)^c^	1.95 (1.54;2.47)^c^	1.44 (1.11;1.88)^c^	1.81 (1.39;2.35)^c^
Habit of having breakfast					
No	1.00	1.00	1.00	1.00	1.00
Yes	1.28 (1.08;1.52)^c^	1.47 (1.18;1.83)^c^	1.25 (0.98;1.59)	1.19 (0.94;1.52)	1.32 (1.03;1.70)^c^
Eating meals in front of screens					
No	1.00	1.00	1.00	1.00	1.00
Yes	0.85 (0.71;1.02)	1.04 (0.80;1.35)	0.89 (0.67;1.17)	0.97 (0.72;1.31)	0.70 (0.54;0.90)^c^
Having meals with the family					
No	1.00	1.00	1.00	1.00	1.00
Yes	1.46 (1.09;1.97)^c^	0.98 (0.73;1.32)	1.12 (0.78;1.61)	1.10 (0.78;1.54)	1.18 (0.82;1.71)

a) PR: Prevalence ratio; b) 95%CI: 95% confidence interval, estimated using Poisson regression adjusted for robust variance; c) P-value < 0.05.

Yellow-Indigenous race/skin color was related to higher regular consumption of beans (PR = 1.41; 95%CI 1.01;1.98) and fruit (PR = 1.78; 95%CI 1.18; 2.67), while those of brown-Black race/color had higher regular consumption of beans (PR = 1.26; 95%CI 1.09;1.46), compared those of White race/skin color. Maternal schooling was positively associated with milk consumption (PR = 1.36; 95%CI 1.06;1.74), and physical exercise was associated with higher milk consumption (PR = 1.41; 95%CI 1.13;1.77), fruit (PR = 1.95; 95%CI 1.54;2.47), raw salads (PR = 1.44; 95%CI 1.11;1.88) and cooked vegetables (PR = 1.81; 95%CI 1.39;2.35). The habit of eating breakfast was associated with the consumption of beans (PR = 1.28; 95%CI 1.08;1.52), milk (PR = 1.47; 95%CI 1.18;1, 83) and cooked vegetables (PR = 1.32; 95%CI 1.03;1.70). Adolescents who reported having meals in front of screens had a 30.0% lower frequency of regular consumption of cooked vegetables (PR = 0.70; 95%CI 0.54;0.90), while those who ate meals with their family reported greater regular consumption of beans (PR = 1.46; 95%CI 1.09;1.97).

The consumption score for healthy eating markers was higher among adolescents who practiced physical activity (PR = 1.30; 95%CI 1.18;1.43), ate breakfast (PR = 1.24; 95% CI 1.13;1.35) and had meals with their family (PR = 1.15; 95%CI 1.01;1.30). In turn, adolescents who ate meals in front of screens had an 11% lower frequency (PR = 0.89; 95%CI 0.81;0.98) for the healthy eating score (Table 4).

**Table 4:** - Crude and adjusted analysis of the score for consumption of healthy eating markers among 9^th^ grade municipal school students (n = 797), Pelotas, Rio Grande do Sul, 2019

Variables	Crude		Adjusted	
	PR^a^ (95%CI^b^)	p-value	PR^a^ (95%CI^b^)	p-value
1^st^ level: demographic variables and socioeconomic variable				
Sex		0.036		0.210
Male	1.00		1.00	
Female	0.92 (0.85;0.99)		0.95 (0.87;1.04)	
Race/skin color		0.043		0.324
Yellow-Indigenous	1.00 (0.92;1.09)		1.00 (0.91;1.10)	
White	1.00		1.00	
Brown/Black	1.28 (1.05;1.56)		1.23 (0.94;1.60)	
Age group (at last birthday)		0.314		0.529
≤ 14	1.00		1.00	
15	1.04 (0.95;1.13)		1.04 (0.95;1.15)	
≥ 16	0.96 (0.86;1.07)		0.98 (0.86;1.12)	
Maternal schooling (completed years of study)		0.607		0.664
≤ 8	1.00		1.00	
9-11	0.99 (0.89;1.09)		0.99 (0.89;1.10)	
≥ 12	1.05 (0.93;1.18)		1.04 (0.93;1.18)	
**2^nd^ level: behavioral variables**				
Physical exercise (minutes/week)		< 0.001		< 0.001
< 300	1.00		1.00	
≥ 300	1.34 (1.22;1.47)		1.30 (1.18;1.43)	
Habit of having breakfast		< 0.001		< 0.001
No	1.00		1.00	
Yes	1.27 (1.17;1.38)		1.24 (1.13;1.35)	
Eating meals in front of screens		0.001		0.018
No	1.00		1.00	
Yes	0.85 (0.77;0.94)		0.89 (0.81;0.98)	
Having meals with the family		0.065		0.032
No	1.00		1.00	
Yes	1.12 (0.99;1.25)		1.15 (1.01;1.30)	

a) PR: Prevalence ratio; b) 95%CI: 95% confidence interval, estimated using Poisson regression adjusted for robust variance.

## DISCUSSION

The results showed that behavioral factors, such as physical exercise, having breakfast, having meals with the family and having meals in front of screens, were strongly related to the adolescents’ food consumption, suggesting that eating habits and behavior are closely associated. Furthermore, it was found that less than three in every 100 adolescents regularly consumed the five foods considered markers of healthy eating.

It should be mentioned that these factors are subject to change and, therefore, seem to influence the process of building a healthy or unhealthy diet.^5^ The 2015 PeNSE survey data also showed that the habits of skipping breakfast, having meals without the company of parents, eating in front of screens or while studying were directly associated with an unhealthy eating pattern.^5^

Physical exercise, for example, is one of the factors associated with better quality of life and health conditions, and is therefore related to the consumption of healthy markers. A study carried out in Minas Gerais found that less active adolescents were more likely to have low consumption of fruit (less than three times a week) and high consumption of soft drinks (more than five times a week).^17^ Similarly, the 2015 PeNSE survey found that prevalence of consumption of ultra-processed foods was higher among adolescents who had had sedentary behavior for a longer period of time.^6^

With regard to dietary patterns, breakfast, a habit was associated with the consumption of three healthy eating markers, is one of the main meals of the day. Having breakfast daily is extremely important, as a positive impact on health, according to evidence found in a study with students who ate breakfast daily and had healthier eating habits, higher consumption of raw vegetables, cooked vegetables, dairy products, fruit, juice fruit and beans.^18^

Having meals with the family positively contributes to the health of young people and is related to greater consumption of foods such as fruit, vegetables, whole grains, beans and dairy products,^19^ lower risk of overweight/obesity and maintenance of healthy eating habits,^20^ as the presence of the family is essential for providing an adequate food environment: the availability of healthy foods and their provision at home is a factor the contributes to the consumption and preference for these foods. This finding is in line with the recommendations of the Food Guide for the Brazilian Population (*Guia Alimentar para a População Brasileira* - GAPB), which emphasizes the positive aspects of the habit of having meals with family members or accompanied by other people.^21^ However, according to the GAPB, the act of eating in front of screens can encourage individuals to eat quickly, without paying full attention to what they are eating, and to prefer ultra-processed foods.^21^

The most recent PeNSE survey took place in 2019, that being same year in which this study was conducted to assess consumption of three markers of healthy eating (fresh fruit; beans; vegetables). Comparison between the PeNSE survey and the results of our study enabled identification of similarities in the consumption of fresh fruit (26.9%), but the PeNSE survey found higher consumption of beans (59.0%) and vegetables (28.8%).^12^ Still in relation to the PeNSE data, with regard to behavioral variables, our study found a higher percentage of having meals with parents, but similarity regarding the frequency of having breakfast.^12^ Milk consumption was not evaluated in the last two editions of the PeNSE survey; however, when analyzed in the 2009 PeNSE survey, milk was the second most consumed marker of healthy eating among students.^22^ In the 2012 PeNSE survey milk consumption was inadequate in around 60.0% of adolescents, whereby it was not consumed at least one day a week.^23^

Less than 3% of the adolescents we interviewed consumed healthy food markers regularly. National surveys have indicated a decline in the consumption of fresh and minimally processed foods (categories in which the studied markers are found) and, on the other hand, an accelerated increase in the consumption of ultra-processed foods.^24^ One of the aspects that has contributed to this scenario has been the lower cost of ultra-processed foods, compared to the cost of fresh foods, such as meat, milk, fruit and vegetables.^25^ This fact may have favored the lower consumption of healthy eating markers among the population of this Pelotas study, i.e. public school students. However, data from the most recent editions of the Household Budget Survey (*Pesquisa de Orçamentos Familiares* - POF) conclude that purchasing of fruit and vegetables throughout Brazil is below the level recommended by the WHO, among all income brackets, showing little variety in the diet of Brazilians.^26^

Another study, also conducted in Pelotas, that compared consumption of ultra-processed foods in three birth cohorts, found higher consumption of ultra-processed foods (33.7% of total energy intake) in the cohort of younger individuals around 11 years old. In the same cohort, higher consumption of these products was found among those with lower income, while in the cohort composed of participants aged 30 years, consumption of them was greater among those with higher income.^27^

Beans, which are traditionally part of the Brazilian diet, have been strongly associated with protection against chronic diseases.^28^ Although it is still the food item with the highest frequency of consumption among the adolescents we assessed, indicating the predominance of traditional food, there is evidence of a tendency of decreasing consumption of beans in the Brazilian population over the last few decades, as shown by the results of the 2013 and 2019 editions of the National Health Survey (*Pesquisa Nacional de Saúde* - PNS).^29^ A bibliographical review^30^ corroborates the findings of the present study, which also revealed association between consumption of beans and being of brown-Black race/skin color, this being a result possibly attributed to the cost of beans.^30^

Rice and beans have a lower cost compared to other healthy eating markers and therefore contribute to maintaining healthy eating practices among the lower socioeconomic strata of the population.^25^ In this research, association between bean consumption and race/skin color was not found in the analysis that assessed the outcome in the form of a food score, which could be explained by the fact that the score aggregates the five healthy foods assessed, thus diluting the relationship between beans and race/skin color. Finally, it is worth noting the positive association between food consumption at school and healthy eating markers, signaling the importance of the actions of the National School Meals Program and the School Health Program for student development and healthy eating habits.^13^

This study has limitations that need to be considered. Self-completion of the questionnaire by students does not allow for a more complex analysis of food consumption, although this type of application is widely used due to its low cost and ease of use.^23^ The time interval defined for obtaining information on food consumption (seven days before the interview) may not have been enough to detect, with greater precision, the eating habits of adolescents. Finally, the use of food consumption markers is limited, as it does not include all foods consumed, even though it is an important tool for monitoring food consumption.^29^

The positive points of this study include the fact that it is a survey of all schools participating in the Health at School Program of the municipal education network in a medium-sized Brazilian city, with a low percentage of losses and refusals. The study also presents a general result for healthy eating markers, despite being stratified for other variables that point to certain groups with a higher risk of low consumption.

This research found a low frequency of regular consumption of the healthy eating markers studied. The behavioral variables exerted a strong influence on the adolescents’ food consumption, indicating that food is not only conditioned to the nutrients present in it, but also to the way, the environment and with whom each adolescent eats, in addition to the behaviors of having breakfast in the morning and being physically active.

The findings of this study can contribute to the targeting of interventions, actions and programs at the local level, aiming to promote food and nutrition education among adolescent students in Pelotas and other populations with similar characteristics, since schools are an ideal space for the promotion of adequate and healthy eating. Since the publication of the NOVA food classification, which intends to guide the food choices of Brazilians in accordance with the Food Guide for the Brazilian Population,^21^ great emphasis has been placed on ultra-processed foods, while little emphasis has been given to the group of fresh or minimally processed foods. Assessment of healthy food consumption is a gap to be explored in the literature.
